# An inventory of greenhouse gas emissions due to natural gas pipeline incidents in the United States and Canada from 1980s to 2021

**DOI:** 10.1038/s41597-023-02177-0

**Published:** 2023-05-13

**Authors:** Hongfang Lu, Zhao-Dong Xu, Y. Frank Cheng, Haoyan Peng, Dongmin Xi, Xinmeng Jiang, Xin Ma, Jun Dai, Yuli Shan

**Affiliations:** 1 China-Pakistan Belt and Road Joint Laboratory on Smart Disaster Prevention of Major Infrastructures, Southeast University, Nanjing, 210096 China; 2 Department of Mechanical Engineering, University of Calgary, Calgary, Alberta T2N 1N4 Canada; 3 School of Science, Southwest University of Science and Technology, Mianyang, 621010 China; 4 School of Geography, Earth and Environmental Sciences, University of Birmingham, Birmingham, B15 2TT UK

**Keywords:** Energy infrastructure, Natural gas

## Abstract

Natural gas is believed to be a critical transitional energy source. However, natural gas pipelines, once failed, will contribute to a large amount of greenhouse gas (GHG) emissions, including methane from uncontrolled natural gas venting and carbon dioxide from flared natural gas. However, the GHG emissions caused by pipeline incidents are not included in the regular inventories, making the counted GHG amount deviate from the reality. This study, for the first time, establishes an inventory framework for GHG emissions including all natural gas pipeline incidents in the two of the largest gas producers and consumers in North America (United States and Canada) from 1980s to 2021. The inventory comprises GHG emissions resulting from gathering and transmission pipeline incidents in a total of 24 states or regions in the United States between 1970 and 2021, local distribution pipeline incidents in 22 states or regions between 1970 and 2021, as well as natural gas pipeline incidents in a total of 7 provinces or regions in Canada between 1979 and 2021. These datasets can improve the accuracy of regular emission inventories by covering more emission sources in the United States and Canada and provide essential information for climate-oriented pipeline integrity management.

## Background & Summary

The United States and Canada are the two largest producers and consumers of natural gas in North America^1–3^. Their natural gas pipeline network is extensive and has been developed over six decades^4–6^. Natural gas pipelines are categorized into three types, namely gathering, transmission, and local distribution pipelines. These categories exhibit notable distinctions in terms of their functions, materials, operating pressures, and other relevant parameters and factors^7^. The detailed information about the three types of natural gas pipelines is listed in Supplementary Table 1. According to statistics in 2021, the mileage of natural gas pipelines in the United States is about 330,000 km, and that in Canada is about 84,000 km^8^. In response to increasing export demands, the construction of natural gas pipelines in the United States and Canada will continue to increase steadily.

Incidents usually occur on natural gas pipelines due to corrosion, external interference, and other relevant reasons during different stages of pipeline operation, from commissioning to their decommissioning^9–11^. The predominant constituent of natural gas is methane. There exists a potential for explosions and consequential casualties in the aftermath of incidents occurring on natural gas pipelines^12–15^. When an incident does not cause combustion, the methane, which has a heating capacity eighty times greater than carbon dioxide, will directly release into the atmosphere^16–18^. Previous studies have analyzed the greenhouse gas (GHG) emissions from natural gas pipelines and equipment, and discussed the impact of installation and operation of natural gas transportation infrastructure on the climate^19–22^. However, relevant inventory works ignored the GHG emissions generated from natural gas pipelines under abnormal conditions (incidents)^23–25^. In fact, although incidents occur occasionally and the resulting GHG emissions account for a relatively small proportion of GHG emissions in regular operations, this part cannot be ignored^26,27^. For example, the incident that occurred in September 2022 on the Nord Stream 1 and 2 natural gas pipelines may have resulted in a release of 220,000 metric tons of methane^28–30^.

To understand the GHG emissions caused by natural gas pipeline incidents in the United States and Canada, this study develops a GHG emissions inventory of natural gas pipeline incidents in the United States and Canada from 1980s to 2021 using Monte Carlo simulation. The dataset is used to show the total GHG emissions from natural gas pipeline incidents in specific states or provinces at a macro level. The developed dataset defines carbon dioxide emissions from natural gas combustion as well as direct methane emissions. In comparison to previous datasets, the dataset developed in this work (1) quantitatively determines the GHG emissions that were not analysed previously; (2) considers and defines the uncertainty associated with the original datasets using Monte Carlo simulations; (3) consider the substantial amount of missing data in the existing datasets from the United States and Canada, and eliminates the analysis limitation; and (4) while the United States’ pre-2010 and Canada’s pre-2008 GHG emissions records were not paid sufficient attention, estimates the values for GHG emissions resulting from natural gas pipeline incidents dating back to the 1980s.

To verify the effectiveness of the proposed method, the estimated results are compared with the GHG emissions using the deterministic method. Based on the GHG emission inventory, both industry and governmental regulators can obtain the risks of GHG emissions caused by natural gas pipeline incidents, and develop a climate-oriented pipeline integrity management plan.

## Methods

### Method for calculating GHG emissions from single-point incidents

After natural gas pipeline incidents, methane may be emitted directly into the atmosphere, or it may enter the atmosphere in the form of carbon dioxide due to combustion or explosion. Both may coexist in the case of partial combustion of natural gas. Additionally, in some incidents, a portion of the residual natural gas remaining in the pipeline needs to be artificially burned for safer maintenance. The aforementioned GHG emissions all belong to the GHG emissions caused by pipeline incidents. Thus, the amount of GHG emissions from a pipeline incident depends primarily on the amount of natural gas released and if the natural gas is combusted by flaring. If the natural gas in the pipeline incident is combusted by flaring, the GHG emissions can be calculated according to Eq. (1)^31^.

1 $${{\rm{GHG}}}_{b}={V}_{1}\times {\rm{AHC}}\times {C}_{c}\times f\times C\times {{\rm{GWP}}}_{n}$$

where GHG_*b*_ is the GHG emissions of natural gas burning, MT CO_2_ eq.; *V*_1_ is the volume of burned natural gas, one thousand cubic feet (Mcf); AHC is the average heat content of natural gas, at 1.036 metric million British thermal unit per thousand cubic feet (mmbtu/Mcf)^31^; *C*_*c*_ is the average carbon coefficient of natural gas burning, at 0.01443 MT carbon/mmbtu^31^; *f* is the fraction of natural gas oxidized into carbon dioxide; *C* is the molecular weight ratio of carbon dioxide to carbon, at 44/12; and GWP_*n*_ is the global warming potential, which equals to 1 for carbon dioxide. Thus, Eq. (1) can be simplified as:

2 $${{\rm{GHG}}}_{b}=0.0548\times {V}_{1}\times f$$

If the incident does not involve combustion of natural gas, the GHG is directly emitted into the atmosphere in the form of methane. The GHG emissions can be calculated according to Eq. (3)^31^. Herein, the methane emissions can be calculated by the value obtained from Eq. (3) divided by GWP_*n*_.

3 $${{\rm{GHG}}}_{ub}=0.028\times \rho \times {V}_{2}\times {{\rm{GWP}}}_{n}$$

where *ρ* is the natural gas density, 0.8 kg/m^3^; *V*_2_ is the volume of natural gas released (without burning) into the atmosphere, Mcf; and GHG_*ub*_ is the GHG emissions resulting from methane, MT CO_2_ eq. As the main component of natural gas is CH_4_, GWP_100_ = 27.9 under the 100-year timeframe from the IPCC AR6 WGI report^31^. Therefore, if the release of natural gas in an incident is defined as *V*, and *V* = *V*_1_ + *V*_2_, the GHG emissions in an incident can be expressed as:

4 $$\begin{array}{ccc}{{\rm{GHG}}}_{total} & = & {{\rm{GHG}}}_{b}+{{\rm{GHG}}}_{ub}=0.0548\times {V}_{1}\times f+0.028\times \rho \times {V}_{2}\times {{\rm{GWP}}}_{n}\\  & = & 0.0548\times V\times \delta \times f+0.028\times \rho \times V\times {{\rm{GWP}}}_{n}\times \left(1-\delta f\right)\end{array}$$

where *δ* is the proportion of burned natural gas in the total released amount in an incident. Additionally, the following assumptions are made when calculating the GHG emissions of a single-point incident^31^:

1. The natural gas release in the incident reported by the operator is accurate.
2. If combustion occurs in the incident, there are 96% to 100% of the burned natural gas is oxidized to carbon dioxide.

### Methodology for estimating GHG emissions of natural gas pipeline incidents at the state-level (or provincial-level)

**Fig. 1:**
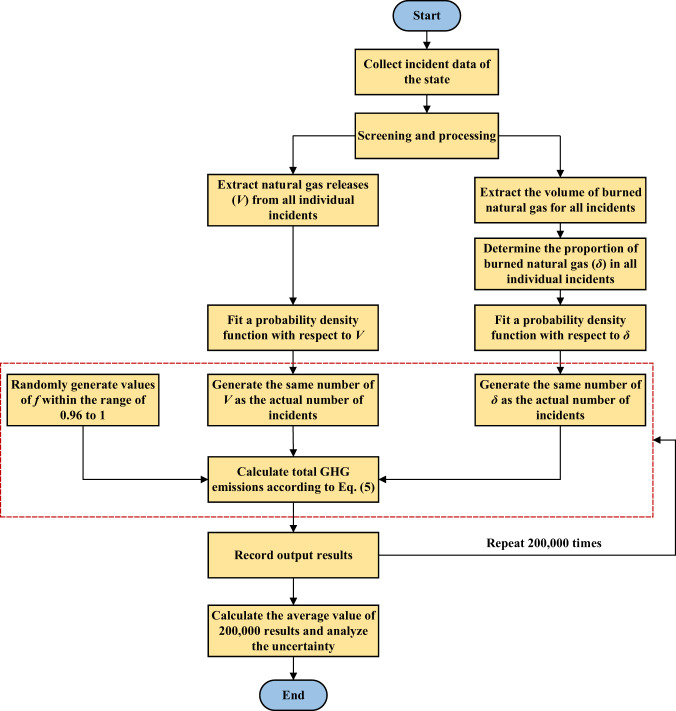
The process for estimating the GHG emissions at the state or provincial level.

Considering the differences in mileages, construction, and management modes of natural gas pipelines in various administrative divisions, it is essential to have an inventory of the GHG emissions caused by natural gas pipeline incidents at the state (or provincial) level. Although some datasets such as PHMSA and CER list the release amount and combustion conditions of natural gas in each pipeline incident, the measurement results are approximate and some data are missing (especially for the CER database). Henceforth, this study utilizes the Monte Carlo simulation technique to estimate the GHG emissions (including carbon dioxide and methane emissions) resulting from natural gas pipeline incidents across various states or provinces within a defined range of uncertainty. The primary principle underlying this methodology involves fitting probability density functions (PDFs) for the released quantity of natural gas and the combustion ratios of natural gas by leveraging existing data, followed by generating multiple points based on these PDFs, equivalent in number to the actual number of incidents, thereby enabling the estimation of GHG emissions. The specific calculation process is shown in Fig. 1. The GHG emissions from all incidents in a state or province are:

5 $${\text{GHG}}_{t}=\mathop{\sum }\limits_{k=1}^{{N}_{t}}{\text{GHG}}_{total,k}$$

where *N*_*t*_ is the total number of incidents in the state or province.

In this study, the PDFs of parameters *V* and *δ* required for estimating GHG emissions from pipeline incidents in the United States were generated based on data from Pipeline & Hazardous Materials Safety Administration (PHMSA) spanning from January 2010 to February 2023. Corresponding PDFs for Canada were generated based on data from Canada Energy Regulator (CER) spanning from January 2008 to February 2023. Based on the obtained PDFs, an equal number of parameters *V* and *δ* were generated, corresponding to the total number of pipeline incidents that occurred between 1970 and 2021 in the United States. This enabled the estimation of GHG emissions resulting from natural gas pipeline incidents in each state of the United States over this 52-year period. A similar approach was used to estimate GHG emissions for Canada spanning from 1979 to 2021 (43-year period). Note that due to the lack of detailed records regarding the release volume and combustion conditions for each incident that occurred in the United States between 1970 and 2009 and in Canada between 1979 and 2007, only information regarding the number of incidents was available for these periods. Hence, the estimated results were based on the assumption that incident characteristics remained unchanged from the 1980s to the present day. The specific description of PHMSA and CER datasets can be found in the section entitled “Data collection”.

The calculation of the proportion of unburned natural gas (methane) in the United States and Canada is slightly different because the CER database only includes the information whether there is combustion in an incident, and does not include the specific volume of natural gas be burned. Therefore, for Canada, the proportion of methane can only be 0 and 1 under the assumption that natural gas is entirely burned or not burned in an incident. Moreover, if the amount of data in the state or province is too small, a significant deviation may be caused when fitting the PDF. Therefore, the data of all states or provinces with an effective data volume of less than 20 are combined to form a new dataset for PDF fitting, as shown in Supplementary Tables 2–4. It is noted that, due to similar operating characteristics for gathering and transmission pipelines and limited amount of data available for gathering pipelines, the GHG emissions during incidents of gathering and transmission pipelines are analyzed together. Actually, the PHMSA also records incidents of the two types of pipelines in the same dataset. Thus, the GHG emissions datasets for gathering & transmission pipeline systems and the local distribution pipeline system are generated in the United States. Moreover, in the Canadian CER dataset, there are only 15 incidents recorded for local distribution pipelines. Thus, the pipeline types cannot be differentiated in analysis of the GHG emissions inventory.

### Data collection

The most comprehensive dataset of natural gas pipeline incidents in the United States is managed by the PHMSA^32^. The PHMSA datasets include all incidents for gathering, transmission, and local distribution pipelines in the United States from 1970 to the present. The PHMSA datasets include all incidents for gathering, transmission, and local distribution pipelines in the United States from 1970 to the present. The incidents of gathering and transmission pipelines are listed in one dataset, while the incidents for local distribution pipelines are included in another dataset. The two datasets record all incident information by time periods from 1970 to mid-1984, from mid-1984 to February 2004, from March 2004 to December 2009, and from 2010 to the present. It is noted that the incident details recorded in the datasets of the four time periods are different. Specifically, the incidents recorded in the dataset from 2010 to the present are described with the most details. Thus, the values of V and δ required for this study are obtained by analysis of this dataset.

The pipeline incident records in Canada are included in the CER dataset^33,34^. Unlike the PHMSA datasets in the United States, the CER dataset documents incident information for all types of pipelines, including gas and liquid pipelines. The CER provides two datasets: i.e., Dataset A containing pipeline incident information from 1979 to the present, and Dataset B containing incident information from 2008 to the present. The latter provides more detailed records of leaked materials and combustion conditions. Nonetheless, the information on leakage in the CER dataset is incomplete. Specifically, in Dataset B, there are a total of 1,735 incidents from January 2008 to February 2023, where only 529 incidents are recorded with information of leakage. Data loss is more substantial in Dataset A, where only 850 out of 3,199 incidents are recorded with leakage information. Supplementary Table 5 lists the differences between the datasets managed by PHMSA and CER.

Upon analysis of the datasets, both the PHMSA and CER datasets suffer from in assessing the environmental impact resulting from the natural gas pipeline incidents. (1) The PHMSA and CER databases do not contain adequate presentations of the GHG emissions. (2) Records from the United States prior to 2010 and from Canada prior to 2008 did not contain quantitative information about the natural gas release volumes, making it difficult, if not impossible, to estimate GHG emissions associated with these incidents. (3) Despite the availability of Canadian natural gas release records since 2008, the substantial amount of missing data disable an effective assessment of GHG emissions resulting from these incidents.

## Data Records

**Fig. 2:**
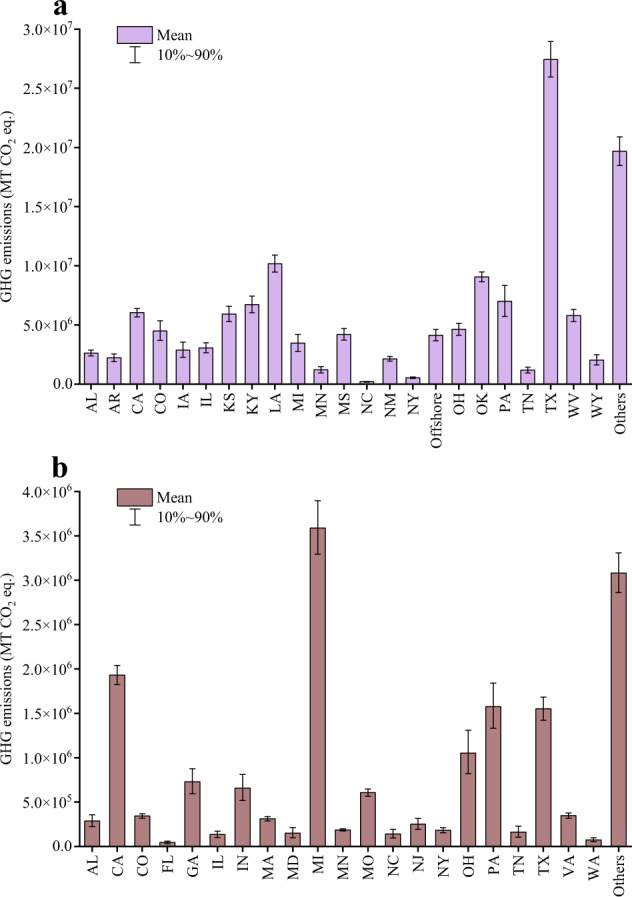
State-level GHG emissions of natural gas pipeline incidents in the United States from 1970 to 2021. (**a**). Gathering and transmission pipelines; (**b**). Local distribution pipelines. Note: The full name of the state name is shown in Supplementary Table 10.

**Fig. 3:**
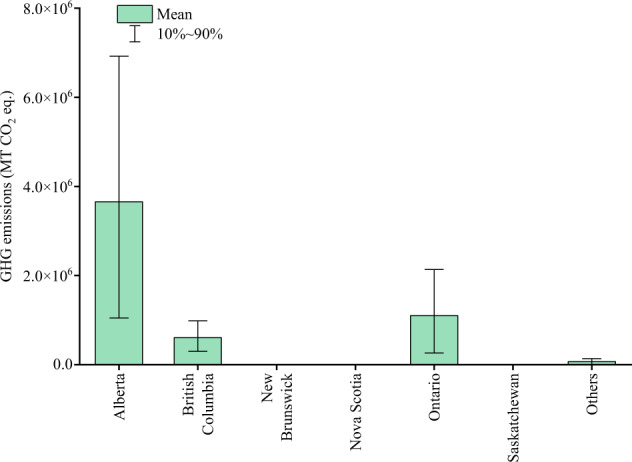
Provincial-level GHG emissions of natural gas pipeline incidents in Canada from 1979 to 2021.

In this study, three datasets were generated^35^, including GHG emissions from gathering and transmission pipeline incidents in the United States between 1970 and 2021 (defined as Dataset 1), GHG emissions from local distribution pipeline incidents in the United States between 1970 and 2021 (defined as Dataset 2), and GHG emissions from Canadian natural gas pipeline incidents between 1979 and 2021 (defined as Dataset 3). Dataset 1 contains 24 states or regions, Dataset 2 contains 22 states or regions, and Dataset 3 contains 7 provinces or regions. Due to the limited data available for some states or provinces, their GHG emissions are merged into the category of “Others”. State-level (or provincial-level) GHG emissions obtained through Monte Carlo simulation are shown in Figs. 2, 3. Specific values and uncertainties are presented in Tables 1–3. According to the Monte Carlo simulation, Texas has the highest GHG emissions from gathering and transmission pipeline systems due to incidents, amounting to (27.44 ± 2.34) million MT CO_2_ eq. In the gas pipeline systems, Michigan has the highest GHG emissions caused by incidents, reaching (3.59 ± 0.47) million MT CO_2_ eq. Alberta in Canada has the highest GHG emissions at (36.55 ± 99.76) million MT CO_2_ eq. Additionally, Supplementary Tables 6–8 and Supplementary Figs. 1, 2 display the carbon dioxide and methane emissions in each system.

**Table 1:** State-level GHG emissions of gathering and transmission pipeline incidents in the United States from 1970 to 2021.

State	Average GHG emissions (MT CO_2_ eq.)	2*σ* (MT CO_2_ eq.)	Uncertainty range
AL	2618926	394498	±15%
AR	2218545	514015	±23%
CA	6035449	560558	±9%
CO	4488816	1302644	±29%
IA	2875526	1005590	±35%
IL	3054093	658042	±22%
KS	5913218	1002228	±17%
KY	6721524	1107477	±16%
LA	10167314	1121977	±11%
MI	3459200	1131079	±33%
MN	1200102	414865	±35%
MS	4196656	763693	±18%
NC	196937	44612	±23%
NM	2129483	306699	±14%
NY	531190	95941	±18%
Offshore	4125304	754526	±18%
OH	4627239	793216	±17%
OK	9060646	663357	±7%
PA	7002348	2041607	±29%
TN	1175842	387922	±33%
TX	27440392	2335346	±9%
WV	5791008	806534	±14%
WY	2030552	681089	±34%
Others	19673854	1902765	±10%

Note: Others include AK, AZ, CT, ID, IN, MA, MD, ME, MO, MT, ND, NE, NJ, NV, SC, SD, UT, VA, WA, and WI. The full names of the state abbreviations are shown in Supplementary Table 9.

**Table 2:** State-level GHG emissions of local distribution pipeline incidents in the United States from 1970 to 2021.

State	Average GHG emissions (MT CO_2_ eq.)	2*σ* (MT CO_2_ eq.)	Uncertainty range
AL	286692	107101	±37%
CA	1930825	168875	±9%
CO	342587	40381	±12%
FL	43948	25057	±57%
GA	728838	225721	±31%
IL	134008	57801	±43%
IN	658157	234966	±36%
MA	311437	37410	±12%
MD	150388	105675	±70%
MI	3589554	474289	±13%
MN	186160	18931	±10%
MO	606344	64441	±11%
NC	140860	81014	±58%
NJ	250542	98659	±39%
NY	181556	48152	±27%
OH	1051651	396768	±38%
PA	1576882	403936	±26%
TN	161728	105502	±65%
TX	1550091	204923	±13%
VA	346950	47332	±14%
WA	73665	33801	±46%
Others	3079607	349861	±11%

Note: Others include AK, AR, CT, DC, DE, HI, IA, ID, KY, LA, MS, MT, ND, NH, NM, OR, RI, SC, SD, UT, WI, WV, WY.

**Table 3:** Provincial-level GHG emissions of natural gas pipeline incidents in Canada from 1979 to 2021.

Province	Average GHG emissions (MT CO_2_ eq.)	2*σ* (MT CO_2_ eq.)	Uncertainty range
Alberta	3654851	9976087	±273%
British Columbia	603889	753406	±125%
New Brunswick	563	1146	±204%
Nova Scotia	176	74	±42%
Ontario	1096593	3674457	±335%
Saskatchewan	4510	2573	±57%
Others	67689	495044	±731%

Note: Others include Manitoba, Northwest Territories, and Quebec.

## Technical Validation

### Uncertainties

In this study, the uncertainty in estimating GHG emissions mainly comes from the operators’ measurements of natural gas release and determination of the combusted amount in incidents. The Monte Carlo simulation results can obtain the uncertainty of each state (or province or region). The uncertainty range is determined by twice the standard deviation (*σ*) of 200,000 Monte Carlo simulation results (These data are available at Figshare^35^). Table 1 displays the uncertainty range of GHG emissions from gathering and transmission pipeline incidents in the United States, which varies between ±7% and ±35%, while the uncertainty range for local distribution pipeline incidents lies between ±9% and ±58% (see Table 2). In comparison to the inventory results for the United States, the uncertainty range for Canada’s inventory results is larger, ranging from ±42% to ±335% (see Table 3).

### Validation of the estimation method

**Fig. 4:**
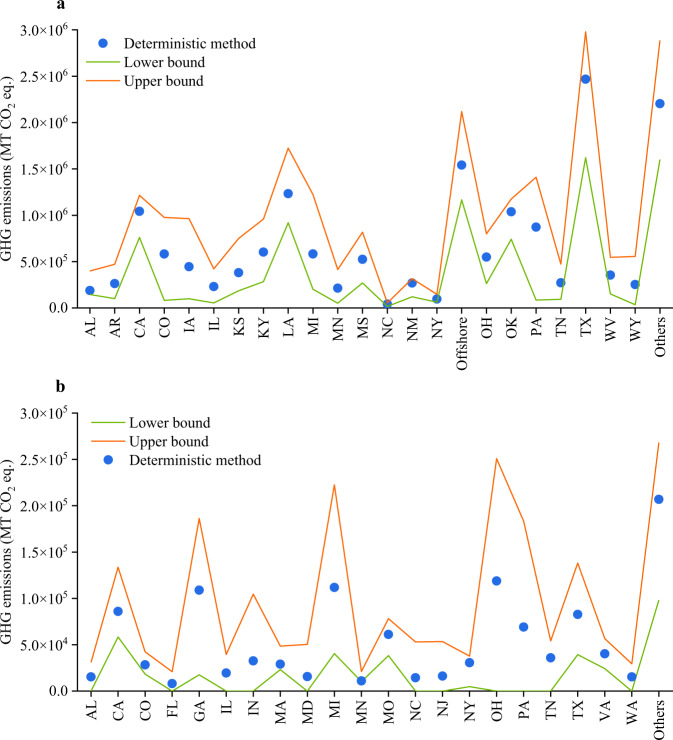
Comparison of the GHG emissions due to natural gas pipeline incidents obtained from two estimation methods. (**a**). Gathering and transmission pipelines in the United States from 2010 to 2021; (**b**). Local distribution pipelines in the United States from 2010 to 2021. Note: Specific data simulated by Monte Carlo are available at Figshare^35^.

To verify the reliability of the GHG emission estimation method proposed in this study, the estimation results were compared with the results of the deterministic method. The deterministic method calculates GHG emissions based on the data provided by single-point incidents and then adds up the GHG emissions caused by all incidents within the respective states (These data are available at Figshare^35^). It is noted that, in calculations of GHG emissions from single-point incidents involving combustion, it is assumed that 98% of natural gas is oxidized to carbon dioxide^36^. Due to the large amount of missing data in the CER dataset, this study used incident data from the United States between 2010 and 2021 for validation. The deviation can be defined as:

6 $$\lambda =\frac{M-D}{D}\times 100 \% $$

where *λ* is the deviation; *M* is the average GHG emissions from Monte Carlo simulations, MT CO_2_ eq.; and *D* is GHG emissions from the deterministic methods, MT CO_2_ eq. Figure 4 illustrates that the GHG emissions estimated using the deterministic approach fall within the range of GHG emissions estimated through the Monte Carlo simulation. Supplementary Tables 10, 11 indicate that the absolute deviation of GHG emissions from gathering and transmission pipeline incidents ranges between 1.79% and 44.42%, while that of local distribution pipelines lies between 0.21% and 49.15%, indicating that the GHG emission estimates derived from the method proposed in this study exhibit a good agreement with the actual values.

## Discussions

This study acknowledges the uncertainty in natural gas release during incidents. However, the accuracy of the GHG emissions (including carbon dioxide and methane emissions) inventory may be affected by incidents that were not reported by operators or remained undetected for a prolonged period. Given the extensive network of pipelines in the United States and Canada, it is not unusual to encounter reporting gaps, missing data, and other issues when dealing with large-scale incident statistics. These challenges are particularly difficult to address in local distribution pipeline systems, where the pipeline locations are usually complicated, making it even harder to identify incidents and accurately measure the natural gas release.

Although the U.S. Environmental Protection Agency (EPA) has made efforts to investigate GHG emissions from natural gas systems^37^, the managed datasets do not specify whether the emissions resulting from incidents are included in the analysis. This occurs because the GHG emissions resulting from pipeline incidents are relatively insignificant when compared to other sources within the natural gas system, such as combustion of natural gas for powering pumps and other electrical equipment. However, incidents like the North Stream pipeline explosion in September 2022 can generate significant GHG emissions. Therefore, a thorough investigation of GHG emissions resulting from pipeline incidents is meaningful for the climate-oriented integrity management of the pipelines. It is recommended that separate inventories be managed to account for GHG emissions resulting from pipeline incidents.

## Supplementary information

Supplementary Information

## Data Availability

The code utilized for the Monte Carlo simulations in this study is provided in the Supplementary Codes 1–3.
