# Supplementary material for: An inventory of greenhouse gas emissions due to natural gas pipeline incidents in the United States and Canada from 1980s to 2021
Source: Sci Data. 2023 May 13;10:282. doi: 10.1038/s41597-023-02177-0 (PMC10183021; doi:10.1038/s41597-023-02177-0)
Supplement: Supplementary file 1 — Supplementary Information [file 41597_2023_2177_MOESM1_ESM.docx]

**Supplementary Information**

*An inventory of greenhouse gas emissions due to natural gas pipeline incidents in the United States and Canada from 1980s to 2021*

*Lu et al.*

**Content**

[**Supplementary Table 1. Classification of natural gas pipeline systems.** 1](#_Toc132989297)

[**Supplementary Table 2. PDFs of *V* and *δ* for gathering and transmission pipeline incidents in the United States.** 2](#_Toc132989298)

[**Supplementary Table 3. PDFs of *V* and *δ* for local distribution pipeline incidents in the United States.** 3](#_Toc132989299)

[**Supplementary Table 4. PDFs of *V* and *δ* for natural gas pipeline incidents in Canada.** 4](#_Toc132989300)

[**Supplementary Table 6. State-level carbon dioxide and methane emissions from incidents of gathering and transmission pipelines in the United States from 1970 to 2021.** 8](#_Toc132989301)

[**Supplementary Table 7. State-level carbon dioxide and methane emissions from incidents of local distribution pipelines in the United States from 1970 to 2021.** 9](#_Toc132989302)

[**Supplementary Table 8. Provincial-level carbon dioxide and methane emissions from incidents of natural gas pipelines in Canada from 1979 to 2021.** 10](#_Toc132989303)

[**Supplementary Table 9. The abbreviation and full name of states in the United States.** 11](#_Toc132989304)

[**Supplementary Table 10. GHG emissions of gathering and transmission pipeline incidents in the United States from 2010 to 2021 using two estimation methods.** 13](#_Toc132989305)

[**Supplementary Table 11. GHG emissions of local distribution pipeline incidents in the United States from 2010 to 2021 using two estimation methods.** 14](#_Toc132989306)

[**Supplementary Fig. 1. State-level carbon dioxide and methane emissions from incidents of natural gas pipelines in the United States from 1970 to 2021. (a) Carbon dioxide emissions for gathering and transmission pipelines. (b) Methane emissions for gathering and transmission pipelines. (c) Carbon dioxide emissions for local distribution pipelines. (b) Methane emissions for local distribution pipelines.** 15](#_Toc132989307)

[**Supplementary Fig. 2. Provincial-level carbon dioxide and methane emissions from incidents of natural gas pipelines in Canada from 1979 to 2021. (a) Carbon dioxide emissions. (b) Methane emissions.** 16](#_Toc132989308)

[**Supplementary Code 1. Python code of Monte Carlo simulation for estimating GHG emissions (or carbon dioxide and methane emissions) in the United States (gathering & transmission pipelines).** 17](#_Toc132989309)

[**Supplementary Code 2. Python code of Monte Carlo simulation for estimating GHG emissions (or carbon dioxide and methane emissions) in the United States (local distribution pipelines).** 32](#_Toc132989310)

[**Supplementary Code 3. Python code of Monte Carlo simulation for estimating GHG emissions (or carbon dioxide and methane emissions) in Canada.** 47](#_Toc132989311)

# **Supplementary Table 1. Classification of natural gas pipeline systems.**

| **Category of natural gas pipelines** | **Gathering pipelines** | **Transmission pipelines** | **Local distribution pipelines** |
| --- | --- | --- | --- |
| Function | Transporting natural gas from wellheads to the treatment plants | Transporting natural gas from treatment plants to city gate stations | Transporting natural gas from city gate stations to users |
| Material | Carbon steels, stainless steels, glass steel | Carbon steels | Carbon steels, polyethylene, composites |
| Diameter (mm) | <450 | 610-1219 | 50-300 |
| Pressure (MPa) | <5 | 1.5-8.5 | <4 |
| Distance | Within several thousand meters | Thousands of kilometers | Thousands of kilometers with a distribution network |
| Inspection requirements | Defect inspection is the primary objective | Defect inspection is the primary objective | Leakage inspection is the primary objective |

# **Supplementary Table 2. PDFs of *V* and *δ* for gathering and transmission pipeline incidents in the United States.**

| **State** | **Number of incidents for PDF fitting from 2010 to Feb 2023** | **Actual number of incidents from 1970 to 2021** | **PDF of *V*** | **PDF of *δ*** |
| --- | --- | --- | --- | --- |
| AL | 24 | 183 | 40 + EXPO(2.4e+04) | WEIB(0.0101, 0.328) |
| AR | 48 | 326 | 4 + WEIB(5.91e+03, 0.524) | 0 |
| CA | 78 | 464 | EXPO(2.15e+04) | WEIB(0.0384, 1.23) |
| CO | 31 | 246 | 384 + WEIB(1.45e+04, 0.494) | POIS(0.0323) |
| IA | 34 | 168 | 192 + WEIB(1.33e+04, 0.561) | 0 |
| IL | 26 | 308 | WEIB(1.03e+04, 0.575) | POIS(0.0385) |
| KS | 42 | 468 | WEIB(1.33e+04, 0.586) | POIS(0.0238) |
| KY | 43 | 412 | 55 + WEIB(1.87e+04, 0.624) | EXPO(0.0305) |
| LA | 175 | 1262 | WEIB(7.82e+03, 0.553) | EXPO(0.0264) |
| MI | 68 | 296 | 0.999 + 3.44e+05 * BETA(0.064, 1.08) | WEIB(0.0303, 0.918) |
| MN | 20 | 103 | 24 + WEIB(1.24e+04, 0.601) | 0 |
| MS | 52 | 371 | 0.999 + WEIB(1.24e+04, 0.603) | EXPO(0.011) |
| NC | 22 | 78 | EXPO(4.04e+03) | 0 |
| NM | 26 | 205 | 2 + EXPO(1.72e+04) | POIS(0.0385) |
| NY | 22 | 122 | 7 + EXPO(6.96e+03) | 0 |
| Offshore | 196 | 485 | WEIB(7.77e+03, 0.54) | 0 |
| OH | 51 | 418 | 5 + WEIB(1.25e+04, 0.612) | POIS(0.0392) |
| OK | 86 | 747 | 10 + EXPO(1.99e+04) | WEIB(0.0274, 0.94) |
| PA | 57 | 450 | 34 + 5.49e+05 * BETA(0.0537, 1.1) | WEIB(0.0257, 0.763) |
| TN | 28 | 112 | 100 + WEIB(1.11e+04, 0.6) | 0 |
| TX | 217 | 2362 | 2.96e+05 * BETA(0.153, 2.21) | WEIB(0.0309, 0.847) |
| WV | 38 | 598 | 14 + WEIB(1.1e+04, 0.616) | WEIB(0.0388, 1.17) |
| WY | 21 | 110 | 224 + WEIB(1.93e+04, 0.595) | LOGN(0.00145, 0.00108) |
| Others | 191 | 1569 | WEIB(1.23e+04, 0.56) | EXPO(0.0172) |

Note: Others include AK, AZ, CT, ID, IN, MA, MD, ME, MO, MT, ND, NE, NJ, NV, SC, SD, UT, VA, WA, and WI.

# **Supplementary Table 3. PDFs of *V* and *δ* for local distribution pipeline incidents in the United States.**

| **State** | **Number of incidents for PDF fitting from 2010 to Feb 2023** | **Actual number of incidents from 1970 to 2021** | **PDF of *V*** | **PDF of *δ*** |
| --- | --- | --- | --- | --- |
| AL | 20 | 490 | WEIB(204, 0.348) | WEIB(0.0271, 0.308) |
| CA | 146 | 3237 | WEIB(425, 0.464) | EXPO(0.0352) |
| CO | 27 | 293 | 0.999 + EXPO(1.92e+03) | WEIB(0.00663, 0.36) |
| FL | 25 | 113 | WEIB(205, 0.41) | WEIB(0.00579, 0.356) |
| GA | 46 | 343 | WEIB(1.22e+03, 0.424) | WEIB(0.00558, 0.356) |
| IL | 40 | 1080 | WEIB(161, 0.361) | WEIB(0.00907, 0.324) |
| IN | 30 | 419 | WEIB(621, 0.368) | WEIB(0.0099, 0.321) |
| MA | 25 | 278 | 0.999 + EXPO(1.86e+03) | EXPO(0.0411) |
| MD | 28 | 288 | WEIB(128, 0.296) | -0.5 + WEIB(0.773, 1.94) |
| MI | 85 | 2321 | WEIB(755, 0.398) | WEIB(0.00625, 0.346) |
| MN | 24 | 415 | EXPO(745) | POIS(0.0417) |
| MO | 35 | 354 | EXPO(2.8e+03) | WEIB(0.0255, 1.25) |
| NC | 24 | 137 | WEIB(459, 0.385) | WEIB(0.00793, 0.349) |
| NJ | 26 | 344 | WEIB(406, 0.376) | -0.5 + WEIB(0.699, 2.06) |
| NY | 98 | 983 | WEIB(60.8, 0.347) | POIS(0.0714) |
| OH | 56 | 535 | WEIB(548, 0.334) | WEIB(0.00752, 0.332) |
| PA | 47 | 843 | WEIB(707, 0.363) | EXPO(0.0542) |
| TN | 21 | 191 | WEIB(229, 0.331) | POIS(0.0476) |
| TX | 97 | 1818 | WEIB(504, 0.428) | WEIB(0.00665, 0.347) |
| VA | 27 | 240 | 1 + EXPO(2.47e+03) | POIS(0.0741) |
| WA | 26 | 142 | WEIB(322, 0.437) | WEIB(0.00661, 0.353) |
| Others | 264 | 4635 | WEIB(231, 0.356) | WEIB(0.00691, 0.345) |

Note: Others include AK, AR, CT, DC, DE, HI, IA, ID, KY, LA, MS, MT, ND, NH, NM, OR, RI, SC, SD, UT, WI, WV, WY.

# **Supplementary Table 4. PDFs of *V* and *δ* for natural gas pipeline incidents in Canada.**

| **Province** | **Number of incidents for PDF fitting from 2008 to Feb 2023** | **Actual number of incidents from 1979 to 2021** | **PDF of *V*** | **PDF of *δ*** |
| --- | --- | --- | --- | --- |
| Alberta | 109 | 259 | WEIB(1.95e+03, 0.179) | POIS(0.0275) |
| British Colombia | 108 | 353 | WEIB(1.5e+03, 0.221) | POIS(0.0556) |
| New Brunswick | 39 | 81 | WEIB(10.5, 0.241) | 0 |
| Nova Scotia | 20 | 23 | EXPO(347) | 0 |
| Ontario | 53 | 267 | WEIB(279, 0.167) | POIS(0.0566) |
| Saskatchewan | 32 | 91 | 2.44e+04 * BETA(0.0307, 0.294) | POIS(0.0313) |
| Others | 19 | 92 | WEIB(23.2, 0.157) | 0 |

Note: Others include Manitoba, Northwest Territories, and Quebec.

**Supplementary Table 5 Information contained in PHMSA and CER datasets.**

| Category | PHMSA dataset | | | | | | | | CER dataset | |
| --- | --- | --- | --- | --- | --- | --- | --- | --- | --- | --- |
|  | Gathering and transmission^32^ | | | | Local distribution^32^ | | | | All types | |
|  | 1970 to mid-1984 | Mid-1984 to 2001 | 2002 to 2009 | 2010 to Feb 2022 | 1970 to mid-1984 | Mid-1984 to Feb 2004 | Mar 2004 to 2009 | 2010 to Feb 2022 | 1979 to Feb 2022^34^ | 2008 to Feb 2022^33^ |
| Operator information | Included | Included | Included | Included | Included | Included | Included | Included | Included | Included |
| Incident information | Included | Included | Included | Included | Included | Included | Included | Included | Included | Included |
| Gas release information | Not included | Not included | Not included | Included | Not included | Not included | Not included | Included | Included but most data are missing | Included but most data are missing |
| Economic loss information | Included | Included | Included | Included | Included | Included | Included | Included | Not included | Not included |
| Pipeline design information | Included | Included | Included | Included | Included | Included | Included | Included | Included | Included |
| Inspection and monitoring information | Not included | Not included | Not included | Included | Not included | Not included | Not included | Included | Included | Not included |
| Incident cause | Included | Included | Included | Included | Included | Included | Included | Included | Not included | Included |
| Drug and alcohol testing information | Not included | Not included | Included | Included | Not included | Not included | Included | Included | Not included | Not included |
| Number of items | 148 | 79 | 196 | 628 | 138 | 62 | 172 | 470 | 97 | 102 |
| Number of incidents recorded | 7864 | 1502 | 1026 | 1617 | 14611 | 2841 | 894 | 1366 | 1210 | 624 |

**Supplementary Table 6. State-level carbon dioxide and methane emissions from incidents of gathering and transmission pipelines in the United States from 1970 to 2021.**

| **State** | **Carbon dioxide emissions** | | | **Methane emissions** | | |
| --- | --- | --- | --- | --- | --- | --- |
|  | **Average emissions (tons)** | **2*σ* (tons)** | **Uncertainty range** | **Average emissions (tons)** | **2*σ* (tons)** | **Uncertainty range** |
| AL | 12502 | 7631 | ±61% | 93420 | 14130 | ±15% |
| AR | 0 | - | - | 79518 | 18423 | ±23% |
| CA | 19237 | 2723 | ±14% | 215635 | 20031 | ±9% |
| CO | 12627 | 22183 | ±176% | 160437 | 46704 | ±29% |
| IA | 0 | - | - | 103065 | 36043 | ±35% |
| IL | 10266 | 12648 | ±123% | 109098 | 23602 | ±22% |
| KS | 12193 | 15184 | ±125% | 211506 | 35939 | ±17% |
| KY | 18115 | 4588 | ±25% | 240266 | 39592 | ±16% |
| LA | 23623 | 3914 | ±17% | 363573 | 40124 | ±11% |
| MI | 9651 | 4826 | ±50% | 123640 | 40432 | ±33% |
| MN | 0 | - | - | 43014 | 14870 | ±35% |
| MS | 9752 | 2699 | ±28% | 150068 | 27311 | ±18% |
| NC | 0 | - | - | 7059 | 1599 | ±23% |
| NM | 7151 | 7201 | ±101% | 76069 | 11023 | ±14% |
| NY | 0 | - | - | 19039 | 3439 | ±18% |
| Offshore | 0 | - | - | 147860 | 27044 | ±18% |
| OH | 15875 | 15645 | ±99% | 165282 | 28453 | ±17% |
| OK | 22511 | 2972 | ±13% | 323948 | 23721 | ±7% |
| PA | 18670 | 9313 | ±50% | 250311 | 72993 | ±29% |
| TN | 0 | - | - | 42145 | 13904 | ±33% |
| TX | 81917 | 11569 | ±14% | 980591 | 83469 | ±9% |
| WV | 18902 | 3712 | ±20% | 206885 | 28817 | ±14% |
| WY | 357 | 182 | ±51% | 72767 | 24408 | ±34% |
| Others | 29534 | 4307 | ±15% | 704097 | 68099 | ±10% |

Note: Others include AK, AZ, CT, ID, IN, MA, MD, ME, MO, MT, ND, NE, NJ, NV, SC, SD, UT, VA, WA, and WI.

**Supplementary Table 7. State-level carbon dioxide and methane emissions from incidents of local distribution pipelines in the United States from 1970 to 2021.**

| **State** | **Carbon dioxide emissions** | | | **Methane emissions** | | |
| --- | --- | --- | --- | --- | --- | --- |
|  | **Average emissions (tons)** | **2*σ* (tons)** | **Uncertainty range** | **Average emissions (tons)** | **2*σ* (tons)** | **Uncertainty range** |
| AL | 3243 | 2847 | ±88% | 10159 | 3823 | ±38% |
| CA | 6031 | 777 | ±13% | 68989 | 6035 | ±9% |
| CO | 875 | 461 | ±53% | 12248 | 1446 | ±12% |
| FL | 102 | 207 | ±203% | 1572 | 897 | ±57% |
| GA | 1629 | 1800 | ±110% | 26065 | 8078 | ±31% |
| IL | 604 | 826 | ±137% | 4782 | 2067 | ±43% |
| IN | 3256 | 3525 | ±108% | 23473 | 8400 | ±36% |
| MA | 1142 | 237 | ±21% | 11122 | 1336 | ±12% |
| MD | 7322 | 5928 | ±81% | 5128 | 3676 | ±72% |
| MI | 9711 | 4457 | ±46% | 128310 | 16969 | ±13% |
| MN | 678 | 462 | ±68% | 6648 | 681 | ±10% |
| MO | 1264 | 204 | ±16% | 21687 | 2305 | ±11% |
| NC | 464 | 878 | ±189% | 5032 | 2898 | ±58% |
| NJ | 9323 | 4456 | ±48% | 8646 | 3447 | ±40% |
| NY | 1146 | 1154 | ±101% | 6466 | 1726 | ±27% |
| OH | 3773 | 4593 | ±122% | 37558 | 14190 | ±38% |
| PA | 7715 | 2846 | ±37% | 56243 | 14411 | ±26% |
| TN | 673 | 2055 | ±305% | 5773 | 3781 | ±65% |
| TX | 4403 | 2028 | ±46% | 55401 | 7331 | ±13% |
| VA | 2274 | 1524 | ±67% | 12354 | 1705 | ±14% |
| WA | 198 | 313 | ±158% | 2633 | 1209 | ±46% |
| Others | 9215 | 3515 | ±38% | 110050 | 12515 | ±11% |

Note: Others include AK, AR, CT, DC, DE, HI, IA, ID, KY, LA, MS, MT, ND, NH, NM, OR, RI, SC, SD, UT, WI, WV, WY.

**Supplementary Table 8. Provincial-level carbon dioxide and methane emissions from incidents of natural gas pipelines in Canada from 1979 to 2021.**

| **Province** | **Carbon dioxide emissions** | | | **Methane emissions** | | |
| --- | --- | --- | --- | --- | --- | --- |
|  | **Average emissions (tons)** | **2*σ* (tons)** | **Uncertainty range** | **Average emissions (tons)** | **2*σ* (tons)** | **Uncertainty range** |
| Alberta | 8547 | 138681 | ±1623% | 130692 | 357531 | ±274% |
| British Columbia | 2956 | 15155 | ±513% | 21539 | 26995 | ±125% |
| New Brunswick | 0 | - | - | 20 | 41 | ±203% |
| Nova Scotia | 0 | - | - | 6 | 3 | ±42% |
| Ontario | 5622 | 83072 | ±1478% | 39103 | 131649 | ±337% |
| Saskatchewan | 12 | 42 | ±342% | 161 | 92 | ±57% |
| Others | 0 | - | - | 2426 | 17744 | ±731% |

Note: Others include Manitoba, Northwest Territories, and Quebec.

**Supplementary Table 9. The abbreviation and full name of states in the United States.**

| **Abbreviation** | **Full name** |
| --- | --- |
| AK | Alaska |
| AL | Alabama |
| AR | Arkansas |
| AZ | Arizona |
| CA | California |
| CO | Colorado |
| CT | Connecticut |
| DC | District of Colombia |
| DE | Delaware |
| FL | Florida |
| GA | Georgia |
| HI | Hawaii |
| IA | Iowa |
| ID | Idaho |
| IL | Illinois |
| IN | Indiana |
| KS | Kansas |
| KY | Kentucky |
| LA | Louisiana |
| MA | Massachusetts |
| MD | Maryland |
| ME | Maine |
| MI | Michigan |
| MN | Minnesota |
| MO | Missouri |
| MS | Mississippi |
| MT | Montana |
| NC | North Carolina |
| ND | North Dakota |
| NE | Nebraska |
| NH | New Hampshire |
| NJ | New Jersey |
| NM | New Mexico |
| NV | Nevada |
| NY | New York |
| OH | Ohio |
| OK | Oklahoma |
| OR | Oregon |
| PA | Pennsylvania |
| RI | Rhode Island |
| SC | South Carolina |
| SD | South Dakota |
| TN | Tennessee |
| TX | Texas |
| UT | Utah |
| VA | Virginia |
| WA | Washington |
| WI | Wisconsin |
| WV | West Virginia |
| WY | Wyoming |

**Supplementary Table 10. GHG emissions of gathering and transmission pipeline incidents in the United States from 2010 to 2021 using two estimation methods.**

| **State** | **Average GHG emissions using Monte Carlo simulation (MT CO_2_ eq.)** | **2*σ* for Monte Carlo simulation (MT CO_2_ eq.)** | **Lower bond for Monte Carlo simulation (MT CO_2_ eq.)** | **Upper bond for Monte Carlo simulation (MT CO_2_ eq.)** | **GHG emissions using deterministic method (MT CO_2_ eq.)** | ***λ* (%)** |
| --- | --- | --- | --- | --- | --- | --- |
| AL | 271902 | 127111 | 144791 | 399013 | 188274 | 44.42 |
| AR | 286011 | 184898 | 101112 | 470909 | 262927 | 8.78 |
| CA | 988379 | 227100 | 761279 | 1215479 | 1042174 | -5.16 |
| CO | 528954 | 447351 | 81602 | 976305 | 582619 | -9.21 |
| IA | 531561 | 432414 | 99147 | 963975 | 445293 | 19.37 |
| IL | 237792 | 183489 | 54303 | 421281 | 230990 | 2.94 |
| KS | 467703 | 282514 | 185189 | 750217 | 380328 | 22.97 |
| KY | 620762 | 337253 | 283509 | 958015 | 602741 | 2.99 |
| LA | 1321464 | 402766 | 918698 | 1724229 | 1233317 | 7.15 |
| MI | 712532 | 510911 | 201621 | 1223443 | 583068 | 22.20 |
| MN | 233130 | 181763 | 51367 | 414893 | 213413 | 9.24 |
| MS | 543571 | 273981 | 269591 | 817552 | 525155 | 3.51 |
| NC | 35338 | 18851 | 16486 | 54189 | 41922 | -15.71 |
| NM | 218190 | 98012 | 120177 | 316202 | 269135 | -18.93 |
| NY | 104521 | 42620 | 61901 | 147142 | 95829 | 9.07 |
| Offshore | 1642675 | 476627 | 1166048 | 2119302 | 1541019 | 6.60 |
| OH | 531287 | 268765 | 262523 | 800052 | 549478 | -3.31 |
| OK | 958169 | 216974 | 741195 | 1175144 | 1037669 | -7.66 |
| PA | 747686 | 662652 | 85033 | 1410338 | 872117 | -14.27 |
| TN | 283298 | 190671 | 92626 | 473969 | 271786 | 4.24% |
| TX | 2300057 | 678475 | 1621582 | 2978532 | 2467764 | -6.80 |
| WV | 348475 | 197538 | 150937 | 546013 | 354891 | -1.81 |
| WY | 295233 | 260690 | 34543 | 555923 | 252841 | 16.77 |
| Others | 2243678 | 643013 | 1600665 | 2886691 | 2204169 | 1.79 |

Note: Others include AK, AZ, CT, ID, IN, MA, MD, ME, MO, MT, ND, NE, NJ, NV, SC, SD, UT, VA, WA, and WI.

**Supplementary Table 11. GHG emissions of local distribution pipeline incidents in the United States from 2010 to 2021 using two estimation methods.**

| **State** | **Average GHG emissions using Monte Carlo simulation (MT CO_2_ eq.)** | **2*σ* for Monte Carlo simulation (MT CO_2_ eq.)** | **Lower bond for Monte Carlo simulation (MT CO_2_ eq.)** | **Upper bond for Monte Carlo simulation (MT CO_2_ eq.)** | **GHG emissions using deterministic method (MT CO_2_ eq.)** | ***λ* (%)** |
| --- | --- | --- | --- | --- | --- | --- |
| AL | 10527 | 20514 | 0 | 31041 | 15373 | -31.52 |
| CA | 96048 | 37619 | 58429 | 133667 | 85951 | 11.75 |
| CO | 30387 | 12011 | 18376 | 42399 | 28370 | 7.11 |
| FL | 9352 | 11587 | 0 | 20939 | 8149 | 14.76 |
| GA | 101892 | 84355 | 17537 | 186248 | 108907 | -6.44 |
| IL | 18248 | 21230 | 0 | 39479 | 19505 | -6.44 |
| IN | 43823 | 60769 | 0 | 104592 | 32589 | 34.47 |
| MA | 35841 | 12673 | 23167 | 48514 | 29091 | 23.20 |
| MD | 15739 | 34600 | 0 | 50339 | 15706 | 0.21 |
| MI | 131478 | 91020 | 40458 | 222498 | 111808 | 17.59 |
| MN | 15691 | 5476 | 10215 | 21166 | 11005 | 42.58 |
| MO | 58231 | 19946 | 38285 | 78177 | 61168 | -4.80 |
| NC | 21525 | 31521 | 0 | 53045 | 14432 | 49.15 |
| NJ | 23277 | 30118 | 0 | 53394 | 16249 | 43.25 |
| NY | 21253 | 16478 | 4774 | 37731 | 30677 | -30.72 |
| OH | 117800 | 132924 | 0 | 250725 | 118806 | -0.85 |
| PA | 88061 | 95230 | 0 | 183291 | 69107 | 27.43 |
| TN | 18586 | 35553 | 0 | 54138 | 35911 | -48.24 |
| TX | 88723 | 49399 | 39324 | 138122 | 82748 | 7.22 |
| VA | 40481 | 16193 | 24287 | 56674 | 40254 | 0.56 |
| WA | 14499 | 15011 | 0 | 29511 | 15360 | -5.61 |
| Others | 183177 | 85024 | 98153 | 268201 | 206827 | -11.43 |

Note: Others include AK, AR, CT, DC, DE, HI, IA, ID, KY, LA, MS, MT, ND, NH, NM, OR, RI, SC, SD, UT, WI, WV, WY.

**Supplementary Fig. 1. State-level carbon dioxide and methane emissions from incidents of natural gas pipelines in the United States from 1970 to 2021. (a) Carbon dioxide emissions for gathering and transmission pipelines. (b) Methane emissions for gathering and transmission pipelines. (c) Carbon dioxide emissions for local distribution pipelines. (b) Methane emissions for local distribution pipelines.**

**Supplementary Fig. 2. Provincial-level carbon dioxide and methane emissions from incidents of natural gas pipelines in Canada from 1979 to 2021. (a) Carbon dioxide emissions. (b) Methane emissions.**

# **Supplementary Code 1. Python code of Monte Carlo simulation for estimating GHG emissions (or carbon dioxide and methane emissions) in the United States (gathering & transmission pipelines).**

from scipy.stats import weibull_min

import numpy as np

import numpy

from sympy.stats import Weibull, density

from sympy import Symbol, pprint

import sympy

import random

import matplotlib.pyplot as plt

import openpyxl

from openpyxl import Workbook

workbook=openpyxl.Workbook()

booksheet1=workbook.create_sheet('AL')

booksheet2=workbook.create_sheet('AR')

booksheet4=workbook.create_sheet('CA')

booksheet5=workbook.create_sheet('CO')

booksheet8=workbook.create_sheet('IA')

booksheet9=workbook.create_sheet('IL')

booksheet11=workbook.create_sheet('KS')

booksheet12=workbook.create_sheet('KY')

booksheet13=workbook.create_sheet('LA')

booksheet16=workbook.create_sheet('MI')

booksheet17=workbook.create_sheet('MN')

booksheet19=workbook.create_sheet('MS')

booksheet20=workbook.create_sheet('NC')

booksheet23=workbook.create_sheet('NM')

booksheet24=workbook.create_sheet('NY')

booksheet25=workbook.create_sheet('offshore')

booksheet26=workbook.create_sheet('OH')

booksheet27=workbook.create_sheet('OK')

booksheet28=workbook.create_sheet('PA')

booksheet29=workbook.create_sheet('TN')

booksheet30=workbook.create_sheet('TX')

booksheet32=workbook.create_sheet('WV')

booksheet33=workbook.create_sheet('WY')

booksheet34=workbook.create_sheet('剩余')

####################################11111

nn=200000

n=183

mean=24000

a=0.0101

b=0.328

AL=np.zeros((nn,2))

for k in range(nn):

sum1=0

sum2=0

for i in range(n):

V= random.expovariate(1/mean)+40

while V<0:

V=random.expovariate(1/mean)+40

x=random.weibullvariate(a, b)

while x<0:

x=random.weibullvariate(a, b)

if x>1:

x=1

sum1=sum1+0.0548*V*x*random.uniform(0.96,1)

sum2=sum2+0.028*0.8*V*(1-x*random.uniform(0.96,1))

AL[k][0]=sum1

AL[k][1]=sum2

####################################22222

n = 326

a=5910

b=0.524

AR=np.zeros((nn,2))

for k in range(nn):

sum1=0

sum2=0

for i in range(n):

V= random.weibullvariate(a, b)+4

while V<0:

V=random.weibullvariate(a, b)+4

x=0

sum1=sum1+0.0548*V*x*random.uniform(0.96,1)

sum2=sum2+0.028*0.8*V*(1-x*random.uniform(0.96,1))

AR[k][0]=sum1

AR[k][1]=sum2

####################################44444

n = 464

mean=21500

a=0.0384

b=1.23

CA=np.zeros((nn,2))

for k in range(nn):

sum1=0

sum2=0

for i in range(n):

V= random.expovariate(1/mean)

while V<0:

V=random.expovariate(1/mean)

x=random.weibullvariate(a, b)

while x<0:

x=random.weibullvariate(a, b)

if x>1:

x=1

sum1=sum1+0.0548*V*x*random.uniform(0.96,1)

sum2=sum2+0.028*0.8*V*(1-x*random.uniform(0.96,1))

CA[k][0]=sum1

CA[k][1]=sum2

####################################55555

n = 246

a=14500

b=0.494

La=0.0323

CO=np.zeros((nn,2))

for k in range(nn):

sum1=0

sum2=0

for i in range(n):

V= random.weibullvariate(a, b)+384

while V<0:

V=random.weibullvariate(a, b)+384

x=np.random.poisson(La)

while x<0:

x=np.random.poisson(La)

if x>1:

x=1

sum1=sum1+0.0548*V*x*random.uniform(0.96,1)

sum2=sum2+0.028*0.8*V*(1-x*random.uniform(0.96,1))

CO[k][0]=sum1

CO[k][1]=sum2

####################################88888

n =168

a=13300

b=0.494

IA=np.zeros((nn,2))

for k in range(nn):

sum1=0

sum2=0

for i in range(n):

V= random.weibullvariate(a, b)+192

while V<0:

V=random.weibullvariate(a, b)+192

x=0

sum1=sum1+0.0548*V*x*random.uniform(0.96,1)

sum2=sum2+0.028*0.8*V*(1-x*random.uniform(0.96,1))

IA[k][0]=sum1

IA[k][1]=sum2

####################################99999

n = 308

a=10300

b=0.575

La=0.0385

IL=np.zeros((nn,2))

for k in range(nn):

sum1=0

sum2=0

for i in range(n):

V= random.weibullvariate(a, b)

while V<0:

V=random.weibullvariate(a, b)

x=np.random.poisson(La)

while x<0:

x=np.random.poisson(La)

if x>1:

x=1

sum1=sum1+0.0548*V*x*random.uniform(0.96,1)

sum2=sum2+0.028*0.8*V*(1-x*random.uniform(0.96,1))

IL[k][0]=sum1

IL[k][1]=sum2

####################################11

n = 468

a=13300

b=0.586

La=0.0238

KS=np.zeros((nn,2))

for k in range(nn):

sum1=0

sum2=0

for i in range(n):

V= random.weibullvariate(a, b)

while V<0:

V=random.weibullvariate(a, b)

x=np.random.poisson(La)

while x<0:

x=np.random.poisson(La)

if x>1:

x=1

sum1=sum1+0.0548*V*x*random.uniform(0.96,1)

sum2=sum2+0.028*0.8*V*(1-x*random.uniform(0.96,1))

KS[k][0]=sum1

KS[k][1]=sum2

####################################12

n = 412

a=18700

b=0.624

mean=0.0305

KY=np.zeros((nn,2))

for k in range(nn):

sum1=0

sum2=0

for i in range(n):

V= random.weibullvariate(a, b)+55

while V<0:

V=random.weibullvariate(a, b)+55

x=random.expovariate(1/mean)

while x<0:

x=random.expovariate(1/mean)

if x>1:

x=1

sum1=sum1+0.0548*V*x*random.uniform(0.96,1)

sum2=sum2+0.028*0.8*V*(1-x*random.uniform(0.96,1))

KY[k][0]=sum1

KY[k][1]=sum2

####################################13

n = 1262

a=7820

b=0.553

mean=0.0264

LA=np.zeros((nn,2))

for k in range(nn):

sum1=0

sum2=0

for i in range(n):

V= random.weibullvariate(a, b)

while V<0:

V=random.weibullvariate(a, b)

x=random.expovariate(1/mean)

while x<0:

x=random.expovariate(1/mean)

if x>1:

x=1

sum1=sum1+0.0548*V*x*random.uniform(0.96,1)

sum2=sum2+0.028*0.8*V*(1-x*random.uniform(0.96,1))

LA[k][0]=sum1

LA[k][1]=sum2

####################################16

n = 296

a1=0.064

b1=1.08

a2=0.0303

b2=0.918

MI=np.zeros((nn,2))

for k in range(nn):

sum1=0

sum2=0

for i in range(n):

V= 344000*random.betavariate(a1,b1)+0.999

while V<0:

V=344000*random.betavariate(a1,b1)+0.999

x=random.weibullvariate(a2, b2)

while x<0:

x=random.weibullvariate(a2, b2)

if x>1:

x=1

sum1=sum1+0.0548*V*x*random.uniform(0.96,1)

sum2=sum2+0.028*0.8*V*(1-x*random.uniform(0.96,1))

MI[k][0]=sum1

MI[k][1]=sum2

####################################17

n = 103

a=12400

b=0.601

MN=np.zeros((nn,2))

for k in range(nn):

sum1=0

sum2=0

for i in range(n):

V= random.weibullvariate(a, b)+24

while V<0:

V=random.weibullvariate(a, b)+24

x=0

sum1=sum1+0.0548*V*x*random.uniform(0.96,1)

sum2=sum2+0.028*0.8*V*(1-x*random.uniform(0.96,1))

MN[k][0]=sum1

MN[k][1]=sum2

####################################19

n = 371

a=12400

b=0.603

MS=np.zeros((nn,2))

for k in range(nn):

sum1=0

sum2=0

for i in range(n):

V= random.weibullvariate(a, b)+0.999

while V<0:

V=random.weibullvariate(a, b)+0.999

x=random.expovariate(1/mean)

while x<0:

x=random.expovariate(1/mean)

if x>1:

x=1

sum1=sum1+0.0548*V*x*random.uniform(0.96,1)

sum2=sum2+0.028*0.8*V*(1-x*random.uniform(0.96,1))

MS[k][0]=sum1

MS[k][1]=sum2

####################################20

n = 78

mean=4040

NC=np.zeros((nn,2))

for k in range(nn):

sum1=0

sum2=0

for i in range(n):

V= random.expovariate(1/mean)

while V<0:

V=random.expovariate(1/mean)

x=0

sum1=sum1+0.0548*V*x*random.uniform(0.96,1)

sum2=sum2+0.028*0.8*V*(1-x*random.uniform(0.96,1))

NC[k][0]=sum1

NC[k][1]=sum2

####################################23

n = 205

mean=17200

La=0.0385

NM=np.zeros((nn,2))

for k in range(nn):

sum1=0

sum2=0

for i in range(n):

V= random.expovariate(1/mean)+2

while V<0:

V=random.expovariate(1/mean)+2

x=np.random.poisson(La)

while x<0:

x=np.random.poisson(La)

if x>1:

x=1

sum1=sum1+0.0548*V*x*random.uniform(0.96,1)

sum2=sum2+0.028*0.8*V*(1-x*random.uniform(0.96,1))

NM[k][0]=sum1

NM[k][1]=sum2

####################################24

n = 122

mean=6960

NY=np.zeros((nn,2))

for k in range(nn):

sum1=0

sum2=0

for i in range(n):

V= random.expovariate(1/mean)+7

while V<0:

V=random.expovariate(1/mean)+7

x=0

sum1=sum1+0.0548*V*x*random.uniform(0.96,1)

sum2=sum2+0.028*0.8*V*(1-x*random.uniform(0.96,1))

NY[k][0]=sum1

NY[k][1]=sum2

####################################25

n = 485

a=7770

b=0.54

offshore=np.zeros((nn,2))

for k in range(nn):

sum1=0

sum2=0

for i in range(n):

V= random.weibullvariate(a, b)

while V<0:

V=random.weibullvariate(a, b)

x=0

sum1=sum1+0.0548*V*x*random.uniform(0.96,1)

sum2=sum2+0.028*0.8*V*(1-x*random.uniform(0.96,1))

offshore[k][0]=sum1

offshore[k][1]=sum2

####################################26

n = 418

a=12500

b=0.612

La=0.0392

OH=np.zeros((nn,2))

for k in range(nn):

sum1=0

sum2=0

for i in range(n):

V= random.weibullvariate(a, b)+5

while V<0:

V=random.weibullvariate(a, b)+5

x=np.random.poisson(La)

while x<0:

x=np.random.poisson(La)

if x>1:

x=1

sum1=sum1+0.0548*V*x*random.uniform(0.96,1)

sum2=sum2+0.028*0.8*V*(1-x*random.uniform(0.96,1))

OH[k][0]=sum1

OH[k][1]=sum2

####################################27

n = 747

mean=19900

a=0.0274

b=0.94

OK=np.zeros((nn,2))

for k in range(nn):

sum1=0

sum2=0

for i in range(n):

V= random.expovariate(1/mean)+10

while V<0:

V=random.expovariate(1/mean)+10

x=random.weibullvariate(a, b)

while x<0:

x=random.weibullvariate(a, b)

if x>1:

x=1

sum1=sum1+0.0548*V*x*random.uniform(0.96,1)

sum2=sum2+0.028*0.8*V*(1-x*random.uniform(0.96,1))

OK[k][0]=sum1

OK[k][1]=sum2

####################################28

n = 450

a1=0.0537

b1=1.1

a2=0.0257

b2=0.763

PA=np.zeros((nn,2))

for k in range(nn):

sum1=0

sum2=0

for i in range(n):

V= 549000*random.betavariate(a1,b1)+34

while V<0:

V=549000*random.betavariate(a1,b1)+34

x=random.weibullvariate(a2, b2)

while x<0:

x=random.weibullvariate(a2, b2)

if x>1:

x=1

sum1=sum1+0.0548*V*x*random.uniform(0.96,1)

sum2=sum2+0.028*0.8*V*(1-x*random.uniform(0.96,1))

PA[k][0]=sum1

PA[k][1]=sum2

####################################29

n = 112

a=11100

b=0.6

TN=np.zeros((nn,2))

for k in range(nn):

sum1=0

sum2=0

for i in range(n):

V= random.weibullvariate(a, b)+100

while V<0:

V=random.weibullvariate(a, b)+100

x=0

sum1=sum1+0.0548*V*x*random.uniform(0.96,1)

sum2=sum2+0.028*0.8*V*(1-x*random.uniform(0.96,1))

TN[k][0]=sum1

TN[k][1]=sum2

####################################30

n = 2362

a1=0.153

b1=2.21

a2=0.0309

b2=0.847

TX=np.zeros((nn,2))

for k in range(nn):

sum1=0

sum2=0

for i in range(n):

V= 296000*random.betavariate(a1,b1)

while V<0:

V=296000*random.betavariate(a1,b1)

x=random.weibullvariate(a2, b2)

while x<0:

x=random.weibullvariate(a2, b2)

if x>1:

x=1

sum1=sum1+0.0548*V*x*random.uniform(0.96,1)

sum2=sum2+0.028*0.8*V*(1-x*random.uniform(0.96,1))

TX[k][0]=sum1

TX[k][1]=sum2

####################################32

n = 598

a1=11000

b1=0.616

a2=0.0388

b2=1.17

WV=np.zeros((nn,2))

for k in range(nn):

sum1=0

sum2=0

for i in range(n):

V= random.weibullvariate(a1, b1)+14

while V<0:

V=random.weibullvariate(a1, b1)+14

x=random.weibullvariate(a2, b2)

while x<0:

x=random.weibullvariate(a2, b2)

if x>1:

x=1

sum1=sum1+0.0548*V*x*random.uniform(0.96,1)

sum2=sum2+0.028*0.8*V*(1-x*random.uniform(0.96,1))

WV[k][0]=sum1

WV[k][1]=sum2

####################################33

n = 110

a=19300

b=0.595

mu=0.00145

sigma=0.00108

WY=np.zeros((nn,2))

for k in range(nn):

sum1=0

sum2=0

for i in range(n):

V= random.weibullvariate(a, b)+224

while V<0:

V=random.weibullvariate(a, b)+224

x=np.random.lognormal(mu,sigma)

while x<0:

x=np.random.lognormal(mu,sigma)

if x>1:

x=1

sum1=sum1+0.0548*V*x*random.uniform(0.96,1)

sum2=sum2+0.028*0.8*V*(1-x*random.uniform(0.96,1))

WY[k][0]=sum1

WY[k][1]=sum2

####################################34

n = 1569

a=12300

b=0.56

mean=0.0172

sy=np.zeros((nn,2))

for k in range(nn):

sum1=0

sum2=0

for i in range(n):

V= random.weibullvariate(a, b)

while V<0:

V=random.weibullvariate(a, b)

x=random.expovariate(1/mean)

while x<0:

x=random.expovariate(1/mean)

if x>1:

x=1

sum1=sum1+0.0548*V*x*random.uniform(0.96,1)

sum2=sum2+0.028*0.8*V*(1-x*random.uniform(0.96,1))

sy[k][0]=sum1

sy[k][1]=sum2

for i in range(1,nn+1):

for j in range(1,3):

booksheet1.cell(i,j).value=AL[i-1][j-1]

booksheet2.cell(i,j).value=AR[i-1][j-1]

booksheet4.cell(i,j).value=CA[i-1][j-1]

booksheet5.cell(i,j).value=CO[i-1][j-1]

booksheet8.cell(i,j).value=IA[i-1][j-1]

booksheet9.cell(i,j).value=IL[i-1][j-1]

booksheet11.cell(i,j).value=KS[i-1][j-1]

booksheet12.cell(i,j).value=KY[i-1][j-1]

booksheet13.cell(i,j).value=LA[i-1][j-1]

booksheet16.cell(i,j).value=MI[i-1][j-1]

booksheet17.cell(i,j).value=MN[i-1][j-1]

booksheet19.cell(i,j).value=MS[i-1][j-1]

booksheet20.cell(i,j).value=NC[i-1][j-1]

booksheet23.cell(i,j).value=NM[i-1][j-1]

booksheet24.cell(i,j).value=NY[i-1][j-1]

booksheet25.cell(i,j).value=offshore[i-1][j-1]

booksheet26.cell(i,j).value=OH[i-1][j-1]

booksheet27.cell(i,j).value=OK[i-1][j-1]

booksheet28.cell(i,j).value=PA[i-1][j-1]

booksheet29.cell(i,j).value=TN[i-1][j-1]

booksheet30.cell(i,j).value=TX[i-1][j-1]

booksheet32.cell(i,j).value=WV[i-1][j-1]

booksheet33.cell(i,j).value=WY[i-1][j-1]

booksheet34.cell(i,j).value=sy[i-1][j-1]

####################################

workbook.save('Gathering and transmission-200000-US.xlsx')

# **Supplementary Code 2. Python code of Monte Carlo simulation for estimating GHG emissions (or carbon dioxide and methane emissions) in the United States (local distribution pipelines).**

from scipy.stats import weibull_min

import numpy as np

import numpy

from sympy.stats import Weibull, density

from sympy import Symbol, pprint

import sympy

import random

import matplotlib.pyplot as plt

import openpyxl

from openpyxl import Workbook

workbook=openpyxl.Workbook()

booksheet1=workbook.create_sheet('AL')

booksheet4=workbook.create_sheet('CA')

booksheet5=workbook.create_sheet('CO')

booksheet6=workbook.create_sheet('FL')

booksheet7=workbook.create_sheet('GA')

booksheet9=workbook.create_sheet('IL')

booksheet10=workbook.create_sheet('IN')

booksheet12=workbook.create_sheet('MA')

booksheet13=workbook.create_sheet('MD')

booksheet16=workbook.create_sheet('MI')

booksheet17=workbook.create_sheet('MN')

booksheet18=workbook.create_sheet('MO')

booksheet19=workbook.create_sheet('NC')

booksheet20=workbook.create_sheet('NJ')

booksheet24=workbook.create_sheet('NY')

booksheet26=workbook.create_sheet('OH')

booksheet28=workbook.create_sheet('PA')

booksheet29=workbook.create_sheet('TN')

booksheet30=workbook.create_sheet('TX')

booksheet32=workbook.create_sheet('VA')

booksheet33=workbook.create_sheet('WA')

booksheet34=workbook.create_sheet('剩余')

####################################11111

nn=200000

n=490

a1=204

b1=0.348

a2=0.0271

b2=0.308

AL=np.zeros((nn,2))

for k in range(nn):

sum1=0

sum2=0

for i in range(n):

V= random.weibullvariate(a1, b1)

while V<0:

V=random.weibullvariate(a1, b1)

x=random.weibullvariate(a2, b2)

while x<0:

x=random.weibullvariate(a2, b2)

if x>1:

x=1

sum1=sum1+0.0548*V*x*random.uniform(0.96,1)

sum2=sum2+0.028*0.8*V*(1-x*random.uniform(0.96,1))

AL[k][0]=sum1

AL[k][1]=sum2

####################################44444

n = 3237

a=425

b=0.464

mean=0.0352

CA=np.zeros((nn,2))

for k in range(nn):

sum1=0

sum2=0

for i in range(n):

V= random.weibullvariate(a, b)

while V<0:

V=random.weibullvariate(a, b)

x=random.expovariate(1/mean)

while x<0:

x=random.expovariate(1/mean)

if x>1:

x=1

sum1=sum1+0.0548*V*x*random.uniform(0.96,1)

sum2=sum2+0.028*0.8*V*(1-x*random.uniform(0.96,1))

CA[k][0]=sum1

CA[k][1]=sum2

####################################55555

n = 293

mean=1920

a=0.00663

b=0.36

CO=np.zeros((nn,2))

for k in range(nn):

sum1=0

sum2=0

for i in range(n):

V= random.expovariate(1/mean)+0.999

while V<0:

V=random.expovariate(1/mean)+0.999

x=random.weibullvariate(a, b)

while x<0:

x=random.weibullvariate(a, b)

if x>1:

x=1

sum1=sum1+0.0548*V*x*random.uniform(0.96,1)

sum2=sum2+0.028*0.8*V*(1-x*random.uniform(0.96,1))

CO[k][0]=sum1

CO[k][1]=sum2

####################################66666

n =113

a1=205

b1=0.41

a2=0.00579

b2=0.356

FL=np.zeros((nn,2))

for k in range(nn):

sum1=0

sum2=0

for i in range(n):

V= random.weibullvariate(a1, b1)

while V<0:

V=random.weibullvariate(a1, b1)

x=random.weibullvariate(a2, b2)

while x<0:

x=random.weibullvariate(a2, b2)

if x>1:

x=1

sum1=sum1+0.0548*V*x*random.uniform(0.96,1)

sum2=sum2+0.028*0.8*V*(1-x*random.uniform(0.96,1))

FL[k][0]=sum1

FL[k][1]=sum2

####################################77777

n =343

a1=1220

b1=0.424

a2=0.00558

b2=0.356

GA=np.zeros((nn,2))

for k in range(nn):

sum1=0

sum2=0

for i in range(n):

V= random.weibullvariate(a1, b1)

while V<0:

V=random.weibullvariate(a1, b1)

x=random.weibullvariate(a2, b2)

while x<0:

x=random.weibullvariate(a2, b2)

if x>1:

x=1

sum1=sum1+0.0548*V*x*random.uniform(0.96,1)

sum2=sum2+0.028*0.8*V*(1-x*random.uniform(0.96,1))

GA[k][0]=sum1

GA[k][1]=sum2

####################################99999

n = 308

a1=161

b1=0.361

a2=0.00907

b2=0.324

IL=np.zeros((nn,2))

for k in range(nn):

sum1=0

sum2=0

for i in range(n):

V= random.weibullvariate(a1, b1)

while V<0:

V=random.weibullvariate(a1, b1)

x=random.weibullvariate(a2, b2)

while x<0:

x=random.weibullvariate(a2, b2)

if x>1:

x=1

sum1=sum1+0.0548*V*x*random.uniform(0.96,1)

sum2=sum2+0.028*0.8*V*(1-x*random.uniform(0.96,1))

IL[k][0]=sum1

IL[k][1]=sum2

####################################10

n = 419

a1=621

b1=0.368

a2=0.0099

b2=0.321

IN=np.zeros((nn,2))

for k in range(nn):

sum1=0

sum2=0

for i in range(n):

V= random.weibullvariate(a1, b1)

while V<0:

V=random.weibullvariate(a1, b1)

x=random.weibullvariate(a2, b2)

while x<0:

x=random.weibullvariate(a2, b2)

if x>1:

x=1

sum1=sum1+0.0548*V*x*random.uniform(0.96,1)

sum2=sum2+0.028*0.8*V*(1-x*random.uniform(0.96,1))

IN[k][0]=sum1

IN[k][1]=sum2

####################################12

n = 278

mean1=1860

mean2=0.0411

MA=np.zeros((nn,2))

for k in range(nn):

sum1=0

sum2=0

for i in range(n):

V= random.expovariate(1/mean1)+0.999

while V<0:

V=random.expovariate(1/mean1)+0.999

x=random.expovariate(1/mean2)

while x<0:

x=random.expovariate(1/mean2)

if x>1:

x=1

sum1=sum1+0.0548*V*x*random.uniform(0.96,1)

sum2=sum2+0.028*0.8*V*(1-x*random.uniform(0.96,1))

MA[k][0]=sum1

MA[k][1]=sum2

####################################13

n = 288

a1=128

b1=0.296

a2=0.773

b2=1.94

MD=np.zeros((nn,2))

for k in range(nn):

sum1=0

sum2=0

for i in range(n):

V= random.weibullvariate(a1, b1)

while V<0:

V=random.weibullvariate(a1, b1)

x=random.weibullvariate(a2, b2)-0.5

while x<0:

x=random.weibullvariate(a2, b2)-0.5

if x>1:

x=1

sum1=sum1+0.0548*V*x*random.uniform(0.96,1)

sum2=sum2+0.028*0.8*V*(1-x*random.uniform(0.96,1))

MD[k][0]=sum1

MD[k][1]=sum2

####################################16

n = 2321

a1=755

b1=0.398

a2=0.00625

b2=0.346

MI=np.zeros((nn,2))

for k in range(nn):

sum1=0

sum2=0

for i in range(n):

V= random.weibullvariate(a1, b1)

while V<0:

V=random.weibullvariate(a1, b1)

x=random.weibullvariate(a2, b2)

while x<0:

x=random.weibullvariate(a2, b2)

if x>1:

x=1

sum1=sum1+0.0548*V*x*random.uniform(0.96,1)

sum2=sum2+0.028*0.8*V*(1-x*random.uniform(0.96,1))

MI[k][0]=sum1

MI[k][1]=sum2

####################################17

n = 415

mean=745

La=0.0417

MN=np.zeros((nn,2))

for k in range(nn):

sum1=0

sum2=0

for i in range(n):

V= random.expovariate(1/mean)

while V<0:

V=random.expovariate(1/mean)

x=np.random.poisson(La)

while x<0:

x=np.random.poisson(La)

if x>1:

x=1

sum1=sum1+0.0548*V*x*random.uniform(0.96,1)

sum2=sum2+0.028*0.8*V*(1-x*random.uniform(0.96,1))

MN[k][0]=sum1

MN[k][1]=sum2

####################################17

n = 354

mean=2800

a=0.0255

b=1.25

MO=np.zeros((nn,2))

for k in range(nn):

sum1=0

sum2=0

for i in range(n):

V= random.expovariate(1/mean)

while V<0:

V=random.expovariate(1/mean)

x=random.weibullvariate(a, b)

while x<0:

x=random.weibullvariate(a, b)

if x>1:

x=1

sum1=sum1+0.0548*V*x*random.uniform(0.96,1)

sum2=sum2+0.028*0.8*V*(1-x*random.uniform(0.96,1))

MO[k][0]=sum1

MO[k][1]=sum2

####################################19

n = 137

a1=459

b1=0.385

a2=0.00793

b2=0.349

NC=np.zeros((nn,2))

for k in range(nn):

sum1=0

sum2=0

for i in range(n):

V= random.weibullvariate(a1, b1)

while V<0:

V=random.weibullvariate(a1, b1)

x=random.weibullvariate(a2, b2)

while x<0:

x=random.weibullvariate(a2, b2)

if x>1:

x=1

sum1=sum1+0.0548*V*x*random.uniform(0.96,1)

sum2=sum2+0.028*0.8*V*(1-x*random.uniform(0.96,1))

NC[k][0]=sum1

NC[k][1]=sum2

####################################20

n = 344

a1=406

b1=0.376

a2=0.699

b2=2.06

NJ=np.zeros((nn,2))

for k in range(nn):

sum1=0

sum2=0

for i in range(n):

V= random.weibullvariate(a1, b1)

while V<0:

V=random.weibullvariate(a1, b1)

x=random.weibullvariate(a2, b2)-0.5

while x<0:

x=random.weibullvariate(a2, b2)-0.5

if x>1:

x=1

sum1=sum1+0.0548*V*x*random.uniform(0.96,1)

sum2=sum2+0.028*0.8*V*(1-x*random.uniform(0.96,1))

NJ[k][0]=sum1

NJ[k][1]=sum2

####################################24

n = 983

a=60.8

b=0.347

La=0.0714

NY=np.zeros((nn,2))

for k in range(nn):

sum1=0

sum2=0

for i in range(n):

V= random.weibullvariate(a, b)

while V<0:

V=random.weibullvariate(a, b)

x=np.random.poisson(La)

while x<0:

x=np.random.poisson(La)

if x>1:

x=1

sum1=sum1+0.0548*V*x*random.uniform(0.96,1)

sum2=sum2+0.028*0.8*V*(1-x*random.uniform(0.96,1))

NY[k][0]=sum1

NY[k][1]=sum2

####################################26

n = 535

a1=548

b1=0.334

a2=0.00752

b2=0.332

OH=np.zeros((nn,2))

for k in range(nn):

sum1=0

sum2=0

for i in range(n):

V= random.weibullvariate(a1, b1)

while V<0:

V=random.weibullvariate(a1, b1)

x=random.weibullvariate(a2, b2)

while x<0:

x=random.weibullvariate(a2, b2)

if x>1:

x=1

sum1=sum1+0.0548*V*x*random.uniform(0.96,1)

sum2=sum2+0.028*0.8*V*(1-x*random.uniform(0.96,1))

OH[k][0]=sum1

OH[k][1]=sum2

####################################28

n = 843

a=707

b=0.363

mean=0.0542

PA=np.zeros((nn,2))

for k in range(nn):

sum1=0

sum2=0

for i in range(n):

V= random.weibullvariate(a, b)

while V<0:

V=random.weibullvariate(a, b)

x=random.expovariate(1/mean)

while x<0:

x=random.expovariate(1/mean)

if x>1:

x=1

sum1=sum1+0.0548*V*x*random.uniform(0.96,1)

sum2=sum2+0.028*0.8*V*(1-x*random.uniform(0.96,1))

PA[k][0]=sum1

PA[k][1]=sum2

####################################29

n = 191

a=229

b=0.331

La=0.0476

TN=np.zeros((nn,2))

for k in range(nn):

sum1=0

sum2=0

for i in range(n):

V= random.weibullvariate(a, b)

while V<0:

V=random.weibullvariate(a, b)

x=np.random.poisson(La)

while x<0:

x=np.random.poisson(La)

if x>1:

x=1

sum1=sum1+0.0548*V*x*random.uniform(0.96,1)

sum2=sum2+0.028*0.8*V*(1-x*random.uniform(0.96,1))

TN[k][0]=sum1

TN[k][1]=sum2

####################################30

n = 1818

a1=504

b1=0.428

a2=0.00665

b2=0.347

TX=np.zeros((nn,2))

for k in range(nn):

sum1=0

sum2=0

for i in range(n):

V= random.weibullvariate(a1, b1)

while V<0:

V=random.weibullvariate(a1, b1)

x=random.weibullvariate(a2, b2)

while x<0:

x=random.weibullvariate(a2, b2)

if x>1:

x=1

sum1=sum1+0.0548*V*x*random.uniform(0.96,1)

sum2=sum2+0.028*0.8*V*(1-x*random.uniform(0.96,1))

TX[k][0]=sum1

TX[k][1]=sum2

####################################32

n = 240

mean=2470

La=0.0741

VA=np.zeros((nn,2))

for k in range(nn):

sum1=0

sum2=0

for i in range(n):

V= random.expovariate(1/mean)+1

while V<0:

V=random.expovariate(1/mean)+1

x=np.random.poisson(La)

while x<0:

x=np.random.poisson(La)

if x>1:

x=1

sum1=sum1+0.0548*V*x*random.uniform(0.96,1)

sum2=sum2+0.028*0.8*V*(1-x*random.uniform(0.96,1))

VA[k][0]=sum1

VA[k][1]=sum2

####################################33

n = 142

a1=322

b1=0.437

a2=0.00661

b2=0.353

WA=np.zeros((nn,2))

for k in range(nn):

sum1=0

sum2=0

for i in range(n):

V= random.weibullvariate(a1, b1)

while V<0:

V=random.weibullvariate(a1, b1)

x=random.weibullvariate(a2, b2)

while x<0:

x=random.weibullvariate(a2, b2)

if x>1:

x=1

sum1=sum1+0.0548*V*x*random.uniform(0.96,1)

sum2=sum2+0.028*0.8*V*(1-x*random.uniform(0.96,1))

WA[k][0]=sum1

WA[k][1]=sum2

####################################34

n = 4635

a1=231

b1=0.356

a2=0.00691

b2=0.345

sy=np.zeros((nn,2))

for k in range(nn):

sum1=0

sum2=0

for i in range(n):

V= random.weibullvariate(a1, b1)

while V<0:

V=random.weibullvariate(a1, b1)

x=random.weibullvariate(a2, b2)

while x<0:

x=random.weibullvariate(a2, b2)

if x>1:

x=1

sum1=sum1+0.0548*V*x*random.uniform(0.96,1)

sum2=sum2+0.028*0.8*V*(1-x*random.uniform(0.96,1))

sy[k][0]=sum1

sy[k][1]=sum2

for i in range(1,nn+1):

for j in range(1,3):

booksheet1.cell(i,j).value=AL[i-1][j-1]

booksheet4.cell(i,j).value=CA[i-1][j-1]

booksheet5.cell(i,j).value=CO[i-1][j-1]

booksheet6.cell(i,j).value=FL[i-1][j-1]

booksheet7.cell(i,j).value=GA[i-1][j-1]

booksheet9.cell(i,j).value=IL[i-1][j-1]

booksheet10.cell(i,j).value=IN[i-1][j-1]

booksheet12.cell(i,j).value=MA[i-1][j-1]

booksheet13.cell(i,j).value=MD[i-1][j-1]

booksheet16.cell(i,j).value=MI[i-1][j-1]

booksheet17.cell(i,j).value=MN[i-1][j-1]

booksheet18.cell(i,j).value=MO[i-1][j-1]

booksheet19.cell(i,j).value=NC[i-1][j-1]

booksheet20.cell(i,j).value=NJ[i-1][j-1]

booksheet24.cell(i,j).value=NY[i-1][j-1]

booksheet26.cell(i,j).value=OH[i-1][j-1]

booksheet28.cell(i,j).value=PA[i-1][j-1]

booksheet29.cell(i,j).value=TN[i-1][j-1]

booksheet30.cell(i,j).value=TX[i-1][j-1]

booksheet32.cell(i,j).value=VA[i-1][j-1]

booksheet33.cell(i,j).value=WA[i-1][j-1]

booksheet34.cell(i,j).value=sy[i-1][j-1]

####################################

workbook.save('Local distribution pipeline-200000-US.xlsx')

# **Supplementary Code 3. Python code of Monte Carlo simulation for estimating GHG emissions (or carbon dioxide and methane emissions) in Canada.**

from scipy.stats import weibull_min

import numpy as np

import numpy

from sympy.stats import Weibull, density

from sympy import Symbol, pprint

import sympy

import random

import matplotlib.pyplot as plt

import openpyxl

from openpyxl import Workbook

nn=200000

n = 259

a=1950

b=0.179

La=0.0275

Albert=np.zeros((nn,2))

for k in range(nn):

sum1=0

sum2=0

for i in range(n):

V= random.weibullvariate(a, b)

while V<0:

V=random.weibullvariate(a, b)

x=np.random.poisson(La)

while x<0:

x=np.random.poisson(La)

if x>1:

x=1

sum1=sum1+0.0548*V*x*random.uniform(0.96,1)

sum2=sum2+0.028*0.8*V*(1-x*random.uniform(0.96,1))

Albert[k][0]=sum1

Albert[k][1]=sum2

####################################

n = 353

a=1500

b=0.221

La=0.0556

BritishColumbia=np.zeros((nn,2))

for k in range(nn):

sum1=0

sum2=0

for i in range(n):

V= random.weibullvariate(a, b)

while V<0:

V=random.weibullvariate(a, b)

x=np.random.poisson(La)

while x<0:

x=np.random.poisson(La)

if x>1:

x=1

sum1=sum1+0.0548*V*x*random.uniform(0.96,1)

sum2=sum2+0.028*0.8*V*(1-x*random.uniform(0.96,1))

BritishColumbia[k][0]=sum1

BritishColumbia[k][1]=sum2

####################################

n = 81

a=10.5

b=0.241

NewBrunswick=np.zeros((nn,2))

for k in range(nn):

sum1=0

sum2=0

for i in range(n):

V= random.weibullvariate(a, b)

while V<0:

V=random.weibullvariate(a, b)

x=0

sum1=sum1+0.0548*V*x*random.uniform(0.96,1)

sum2=sum2+0.028*0.8*V*(1-x*random.uniform(0.96,1))

NewBrunswick[k][0]=sum1

NewBrunswick[k][1]=sum2

####################################

n = 23

mean=347

a=10.5

b=0.241

NovaScotia=np.zeros((nn,2))

for k in range(nn):

sum1=0

sum2=0

for i in range(n):

V= random.expovariate(1/mean)

while V<0:

V=random.expovariate(1/mean)

x=0

sum1=sum1+0.0548*V*x*random.uniform(0.96,1)

sum2=sum2+0.028*0.8*V*(1-x*random.uniform(0.96,1))

NovaScotia[k][0]=sum1

NovaScotia[k][1]=sum2

####################################

n = 267

a=279

b=0.167

La=0.0566

Ontario=np.zeros((nn,2))

for k in range(nn):

sum1=0

sum2=0

for i in range(n):

V= random.weibullvariate(a, b)

while V<0:

V=random.weibullvariate(a, b)

x=np.random.poisson(La)

while x<0:

x=np.random.poisson(La)

if x>1:

x=1

sum1=sum1+0.0548*V*x*random.uniform(0.96,1)

sum2=sum2+0.028*0.8*V*(1-x*random.uniform(0.96,1))

Ontario[k][0]=sum1

Ontario[k][1]=sum2

####################################

n = 91

a=0.0307

b=0.294

La=0.0313

Saskatchewan=np.zeros((nn,2))

for k in range(nn):

sum1=0

sum2=0

for i in range(n):

V= 24400*random.betavariate(a,b)

while V<0:

V= 24400*random.betavariate(a,b)

x=np.random.poisson(La)

while x<0:

x=np.random.poisson(La)

if x>1:

x=1

sum1=sum1+0.0548*V*x*random.uniform(0.96,1)

sum2=sum2+0.028*0.8*V*(1-x*random.uniform(0.96,1))

Saskatchewan[k][0]=sum1

Saskatchewan[k][1]=sum2

####################################

n = 92

a=23.2

b=0.157

sy=np.zeros((nn,2))

for k in range(nn):

sum1=0

sum2=0

for i in range(n):

V= random.weibullvariate(a, b)

while V<0:

V= random.weibullvariate(a, b)

x=0

sum1=sum1+0.0548*V*x*random.uniform(0.96,1)

sum2=sum2+0.028*0.8*V*(1-x*random.uniform(0.96,1))

sy[k][0]=sum1

sy[k][1]=sum2

####################################

workbook=openpyxl.Workbook()

booksheet1=workbook.create_sheet('Albert')

booksheet2=workbook.create_sheet('BritishColumbia')

booksheet3=workbook.create_sheet('NewBrunswick')

booksheet4=workbook.create_sheet('NovaScotia')

booksheet5=workbook.create_sheet('Ontario')

booksheet6=workbook.create_sheet('Saskatchewan')

booksheet7=workbook.create_sheet('sy')

for i in range(1,nn+1):

for j in range(1,3):

booksheet1.cell(i,j).value=Albert[i-1][j-1]

booksheet2.cell(i,j).value=BritishColumbia[i-1][j-1]

booksheet3.cell(i,j).value=NewBrunswick[i-1][j-1]

booksheet4.cell(i,j).value=NovaScotia[i-1][j-1]

booksheet5.cell(i,j).value=Ontario[i-1][j-1]

booksheet6.cell(i,j).value=Saskatchewan[i-1][j-1]

booksheet7.cell(i,j).value=sy[i-1][j-1]

workbook.save('200000-Canada.xlsx')
